# Bin-Picking for Planar Objects Based on a Deep Learning Network: A Case Study of USB Packs

**DOI:** 10.3390/s19163602

**Published:** 2019-08-19

**Authors:** Tuan-Tang Le, Chyi-Yeu Lin

**Affiliations:** 1 Department of Mechanical Engineering, National Taiwan University of Science and Technology, Taipei 106, Taiwan; 2 Taiwan Building Technology Center, National Taiwan University of Science and Technology, Taipei 106, Taiwan; 3 Center for Cyber-Physical System, National Taiwan University of Science and Technology, Taipei 106, Taiwan

**Keywords:** random bin-picking, instance segmentation, object detection, pose estimation, artificial intelligence, neural network

## Abstract

Random bin-picking is a prominent, useful, and challenging industrial robotics application. However, many industrial and real-world objects are planar and have oriented surface points that are not sufficiently compact and discriminative for those methods using geometry information, especially depth discontinuities. This study solves the above-mentioned problems by proposing a novel and robust solution for random bin-picking for planar objects in a cluttered environment. Different from other research that has mainly focused on 3D information, this study first applies an instance segmentation-based deep learning approach using 2D image data for classifying and localizing the target object while generating a mask for each instance. The presented approach, moreover, serves as a pioneering method to extract 3D point cloud data based on 2D pixel values for building the appropriate coordinate system on the planar object plane. The experimental results showed that the proposed method reached an accuracy rate of 100% for classifying two-sided objects in the unseen dataset, and 3D appropriate pose prediction was highly effective, with average translation and rotation errors less than 0.23 cm and 2.26°, respectively. Finally, the system success rate for picking up objects was over 99% at an average processing time of 0.9 s per step, fast enough for continuous robotic operation without interruption. This showed a promising higher successful pickup rate compared to previous approaches to random bin-picking problems. Successful implementation of the proposed approach for USB packs provides a solid basis for other planar objects in a cluttered environment. With remarkable precision and efficiency, this study shows significant commercialization potential.

## 1. Introduction

Industrial automation technology has been evolving for a long time and is constantly being improved to increase productivity and gradually reduce the need for direct human intervention. One of the fundamental goals of robotic research is to build a system that is smarter and more flexible and has the ability to work independently. Such a system would be able to completely replace people in the implementation of certain tasks, including those in the field of automated manufacturing. One of the main tasks in the production line that still requires human execution is random bin-picking, which includes observing and computing object poses [1]. A smart system can replace the manual labor required to interact with the surrounding environment. This means possessing the ability to understand the environment and address uncertain situations by taking appropriate action [2]. However, achieving satisfactory perceptual ability for a complete and optimal robot bin-picking system remains a challenging and complex issue because most parts come in randomly located boxes or bins and there are vast differences in the geometric shape, color, texture, and surface of objects to be processed on different production lines. It is also important to note that the system must be able to work in situations in which objects are randomly located in an unstructured and poorly constrained occlusion in a heavily cluttered environment. Random bin-picking has been the focus of research for many years because of its necessity and high applicability in the industry, and it remains a core topic for improvement in the field of image processing and automated manufacturing. However, because studies have either been based on a simplistic hypothesis or have been insufficiently robust for industries with strict requirements for stability and speed, most research results have been somewhat limited [3]. It is therefore necessary to continue to develop algorithms and build a complete system to overcome challenges in industrial bin-picking.

Although an increasing number of robots are being designed to help people perform labor-intensive tasks that need to be performed repeatedly and accurately or in dangerous environments, there are still several daily challenges that robots face [4]. Because random bin-picking is the final target system, one of the most challenging tasks overall is grasping the object in the right position with the correct orientation in an unbounded environment once it has been located in 3D space. Given the importance of the module in the overall process, so-called pose estimation is a key function, not only for efficient and flexible object inspection but also for the grasping or manipulation system [5]. Pose estimation, which originated in the early 1960s, is a mainstream component of the latest two directions pursued in modern computer vision research. This research, which has been developing for more than 50 years, has passed through each stage of development to gradually achieve four corresponding levels of computer vision: detection, localization, recognition, and scene understanding [6]. Thanks to an increase in computational power and the development of 3D sensor technologies [7], object recognition has demonstrated remarkable achievements and has created new approaches and research directions that can solve complex problems that have had no effective solution in the past. The most well-known lines of research in the field of object pose estimation are feature-based, template matching, and machine learning or deep learning methods.

The most extensive solution to the problem of object recognition is employing feature-based methods that utilize three-dimensional data [5]: 3D information can easily be retrieved using inexpensive contact-free sensors (such as cameras) that are essential for continuous and fast calculation [2]. There are two approaches based on feature-based methods: local and global. Local feature-based methods [8] usually extract information around selected key points based on matching descriptors of local surface features, whereas global feature-based methods employ a different pipeline for which a single or small set of descriptors describes the entire surface of the object (e.g., Refs. [9,10,11,12]).

Template matching techniques are the second most common solution, and these directly use RGB images or extend from two-dimensional computer vision to RGB-D data to predict object poses. Image gradients [13,14] are the key to this approach and have yielded relatively good results due to changes in occlusion, clutter, and illumination in 2D images. Based on previous research results, Hinterstoisser et al. [15] proposed extending the template matching technique to RGB-D data as a new approach to quantized surface normal as a depth cue. In the same fashion, Ulrich et al. [16] only used an image for fast 3D object recognition by combining scale space and similarity-based aspect graphs. A 3D CAD model of the object with geometry information helped to generate a hierarchical model for the approach without relying on texture or reflectance information. Ye et al. [17] proposed a real-time pose estimation of texture-less objects using hierarchical search strategy-based template matching through a template pyramid, which was 44 times faster than the original search method. Another hierarchical detection method using template matching in combination with the physical characteristics of the object was proposed by Su et al. [18]. This allowed the pose of the object to be estimated in an occluding situation. Muñoz et al. [19] developed a 6D pose estimation algorithm using a single RGB image that combined template matching and a part-based method called cascaded forests templates.

Supervised machine learning [20,21] and recent techniques based on deep neural networks [22] have also been used for pattern recognition and pose estimation. RGB-D data are the common input in computer vision, including in machine learning techniques. Blum et al. [23] introduced a learned feature descriptor for object recognition using RGB-D images based on advances in the machine learning technique known as the convolutional *k*-means descriptor. One of the popular types of geometric data structures is the point cloud. In a recent study by Qi et al. [24], their neural network could directly use point cloud data without transforming them into regular 3D voxel grids or image collections. This neural network is called PointNet and serves as the pioneer in this direction. However, PointNet lacks the ability to capture local structure information, leading to difficulties in recognizing fine-grained patterns and generalizability in complex scenes. An improved version of PointNet, which is known as PointNet++ [25], solves the above bottleneck with the ability to learn deep point set features efficiently and robustly. Brachmann et al. [26] proposed an object pose estimation method for both textured and texture-less objects based on random forests. This method classifies each pixel as a continuous coordinate on a canonical body in a canonical pose before applying geometric refinement in the next stage. Brachmann et al. [27] continued to advance the 3D pose estimation technique to a new level using only a single RGB image. Employing the same approach, Do et al. [28] recently predicted object poses by extending instance segmentation networks with a novel pose estimation branch to directly regress 6D object poses without any postrefinements. This is sufficiently accurate and quick for robotics applications.

In addition to studies focusing on estimating object poses, a few studies have also focused on complete random bin-picking systems. CAD-based pose estimation is one of the most popular approaches to solving random bin-picking problems. Liu et al. [2] proposed a CAD-based method for successfully creating a practical vision-based bin-picking robotic system capable of detecting and estimating the pose of an object before performing error detection and pose correction while the part is in the gripper. In this approach, a multiflash camera is used to extract robust depth edges. The overall detection rate was reported to be 95% with a grasping success rate of 94% and a processing time of approximately 1.9 s per object. The CAD model with an RGB-D camera is a strong combination for solving random bin-picking problems [29,30] in which multiple objects are presented in a cluttered environment. Wu et al. [29] proposed a system using a Kinect RGB-D sensor that achieved a 93.9% average recognition rate of three different types of objects and could pick up the object with a success rate of 89.7%. This is a method used to reduce the number of 3D point cloud candidates, while another filter was designed to remove less accurate matching and occluded poses to increase the success of the picking rate. Chen et al. [30] used two depth cameras to acquire all of the necessary features of the objects and proposed a CAD-based multiview pose estimation algorithm with noise removal and an object segmentation module. A complete system was designed for the pick-and-place task based on structured light cameras, 3D pose estimation, and robot arm control.

As an alternative to the CAD-based model, 3D reconstruction can also be used for pose estimation. For instance, a vision system consisting of a camera and a laser projector placed on the arm to reconstruct the target object’s 3D point cloud was introduced by Chang et al. [1]. This has potential applications in production lines. Few studies have utilized physical information concerning the object and surface as the main feature to create a robust system that can execute random bin-picking tasks. Martinez et al. [3] developed an automatic bin-picking system that provides a complete and robust solution. In their study, useful edge information was used for the recognition part, and 3D surface information was used to calculate the location of the object in the scene.

Among the existing model-based approaches, one of the most successful 6D pose estimation methods is the point pair feature [31], an integrated and compromised alternative to traditional local and global pipelines [32]. Vidal et al. [32] presented an improved method based on point pair features and an extension [5] for free-form rigid 6D pose estimation. A method based on a voting-based approach for pose estimation was proposed by Choi et al. [33]. This work extended the PPF for applying planar objects by using boundary points with directions and boundary line segments besides oriented surface points. Feature-based template matching has also been combined with machine learning and deep learning techniques. These approaches have produced encouraging results and have become a key research trend in recent years. Spenrath et al. [34] developed a method comprising several neural networks for heuristic search algorithms to reduce the calculation time and allow networks to learn from the properties of objects, increasing the likelihood of finding a good position from which to grasp the object. Lin et al. [4] recently published impressive results on visual object recognition and pose estimation based on deep semantic segmentation networks.

Given its crucial role in the fields of industrial manipulation, logistics classification, and household cleaning, random object picking that provides flexibility and high efficiency has attracted considerable attention [3]. However, it is difficult to find systems that can implement random bin-picking tasks for planar objects in a cluttered environment, especially thin objects. Regarding the physical structure of objects, planar objects seem to be considered simpler for random bin-picking tasks than free-form objects. Nevertheless, many industrial and real-world objects are planar items whose oriented surface points are not sufficiently compact and discriminative [33]. Thus, the methods that mainly use geometry information, especially depth discontinuities, cannot work well for planar objects.

Over the past few years, deep learning-based object recognition algorithms have shown very promising results in the field of robotic vision applications. Therefore, in this study, we utilized the pick-and-place task for planar objects in a cluttered environment, especially thin ones, by combining an instance segmentation-based deep learning approach with a novel method to predict the appropriate pose for picking up objects. This paper presents details of the entire system structure, along with its implementation and verification.

The paper presents a novel and robust random bin-picking system for planar objects, especially thin objects, which lack geometry information, in a cluttered environment. The complete system fully integrates a state-of-the-art instance segmentation network with a new method for building the appropriate coordinate system on the target object. In detail, the contribution of this work is a novel approach to a planar objects random bin-picking system. The proposed system inherits powerful object recognition from the initial stage. This method is a global solution with high efficiency to overcome the challenge of oriented surface points on planar objects, as above mentioned. In contrast to another approach, the proposed system initiates from 2D image data instead of 3D information. This approach does not mainly rely on depth discontinuities, which is crucial in other methods (e.g., the point pair feature approach [5,31,32] or CAD-based pose estimation approach [2,29,30]). The successful implementation also demonstrated an effective technique to find correspondence between the RGB and depth image of the Kinect v2. In addition, the technique for taking the ground truth of the object with respect to the robot base serves as a pioneer method for evaluating the overall rotation and rotation accuracy of the whole system. To the best of our knowledge, the highest successful pick-up rate in the previous works for random bin-picking tasks was 98%. Our proposed system, nonetheless, achieved an overall successful pickup rate of over 99% at an average processing time of 0.9 s per step. This is fast enough for robotic continuous operation without interruption. This research thus shows impressive results in term of accuracy.

The remainder of this paper is organized as follows. Section 2 provides an overview of the vision equipment used in the research. It also presents a brief introduction to the image processing part and the prediction of six poses of the target objects before going on to consider the industrial manipulator for object picking. The proposed deep learning algorithm, including data creation, data augmentation, training, and testing, is introduced in Section 3. Section 4 discusses the proper 3D pose prediction in detail, and the experimental results for each module are presented and discussed in Section 5. Finally, conclusions and future work are discussed in Section 6.

## 2. System Architecture

One of the most intriguing and challenging industrial robotics applications is random bin-picking in a cluttered environment. This system is complicated because it is an autonomous system that encompasses all integration and interactions among the visual perception system, robot operation, and control system. An independent system such as this that controls every situation can replace people in simple and complex tasks. A system performing such tasks requires a visual ability to observe objects in the scene before bringing this information to the processor. The information is processed in a manner that is similar to the analytical processing of the human brain, which orders limbs to perform tasks. Instead of people using limbs, this system uses different actuators to perform tasks depending on their complexity, such as simple mechanical mechanisms, parallel robots, or industrial manipulators.

**Figure 1:**
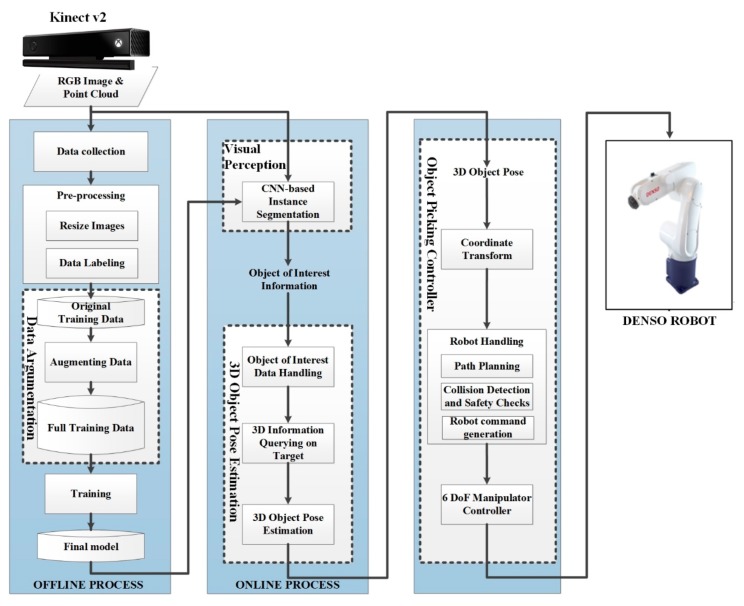
Architecture of the proposed system.

In this novel automated system for a planar object approach, a depth sensor (Kinect v2, Microsoft Corporation, Redmond, WA, USA) is used to retrieve 2D information used for image processing and extract 3D information used to perform picking tasks at the final stage of each cycle. Here, 2D information is processed by a deep learning network to detect and locate the objects in a heavily cluttered environment before it is sent to the next module to predict the 3D pose of the objects. Finally, an industrial manipulator (Denso 6556, Denso Corporation, Kariya, Japan) is used to perform the actual task of picking up objects. The architecture of the proposed system is presented in Figure 1.

### 2.1. Kinect Sensor

Kinect is an RGB-D sensor capable of simultaneously providing both an RGB color image and a depth image. Microsoft introduced the first version in 2010 as a game device for the Xbox 360 platform (Microsoft Corporation, Redmond, WA, USA). In 2012, a second version called “Kinect for Windows” or “Kinect v2” was released, not only for gaming but also for commercial use (primarily in the field of computer vision). Although the first version of the Kinect (v1) (Microsoft Corporation, Redmond, WA, USA) had poor RGB color image resolution (640 × 480 pixels), the new version (Kinect v2) provides very high RGB color image resolution (1920 × 1080 pixels). Table 1 shows details of the image resolution for both versions of the Kinect. This feature is extremely beneficial in enabling color image information to be used to implement image processing. Recently, Amit et al. [35] showed that the Kinect v1 can be used for highly demanding precision operations and complex manufacturing tasks. In addition, many studies have also indicated that the second generation of Kinect sensors has advantages over the first version in terms of performance, accuracy, and a wider field of view (FOV) [36,37]. These characteristics made the Kinect v2 sensor a good choice for the proposed method. However, any type of 3D camera with similar or better characteristics can be used. Any industrial 3D sensors that meet the initial conditions can replace Kinect v2 for better results. In this research, Kinect v2 was handily chosen to fulfill our overall approach.

### 2.2. Recognition and Appropriate Pose Estimation

The proposed vision-based object detection and pose estimation algorithm consists of four main modules: data augmentation to create a common object in context (COCO) style dataset, visual perception, 3D pose estimation, and a target picking controller and actuator. The visual perception module, a deep neural network known as a semantic segmentation algorithm, is used to recognize the objects in a heavily cluttered environment. The quality of input training data used to optimize deep learning models is one of the major determinants of model quality. To build a model that works well in a cluttered environment with uncertain randomness, a module was added to the offline section so that it can easily create training data with the right format and enrich data under the control of a small dataset without creating any noise. Details of the proposed visual perception and data augmentation methods are presented in the implementation section.

Forcing the network to learn how to detect only the best objects-of-interest (OOIs) in a scene means the trained visual perception module is used in the online section to detect and recognize only the best OOIs at the pixel level. Another module, OOI data handling (OOI-DH), is used to handle all random and coarse data from the object recognition module. One of the challenges in this approach is mapping between 2D pixels in an RGB image to the same position in a depth image. We proposed that this could be achieved using a linear regression model: 3D information is then easily extracted using the libfreenect2 library. This 3D information is the basis for the next module, which estimates the appropriate 3D pose to pick up objects. In this estimation, a module takes the next step by determining the best target object handling (BTOH): this handles the entire procedure, selects only the best target, and prepares all of the information needed to predict the proper 3D pose for further steps. The final 3D pose prediction contains two parts, translation and rotation, which are estimated and optimized using two different approaches. The details of the proposed 3D pose estimation are discussed in Section 4. The final module is a six degrees of freedom industrial robot arm used to pick up objects in real scenarios based on the predicted object 3D pose. The aim of this study was to provide a complete bin-picking solution for industrial problems on planar objects: we discuss this in detail as follows and discuss its actual performance on our target picking controller.

### 2.3. Object Picking Controller

An industrial manipulator (Denso 6556) was used to operate the final step of the proposed autonomous system by picking up objects. This industrial manipulator is a small-sized, vertical articulated model with six degrees of freedom that drives the arms to assemble and transport workpieces with the motors. The end effector was unavailable. Therefore, for the picking task, a vacuum suction-type end effector was implemented. To achieve acceptable and smooth performance, the path planning step focused on checking the work area and the posture of the robot to ensure that all parameters were optimal before operation was planned in conjunction with safety handling.

## 3. Proposed Deep Learning Algorithm

### 3.1. Surpervised Learning Approach

One of the most popular directions in deep learning research is supervised learning, an algorithm that analyzes the training data and produces an inferred function that maps an input to an output based on example input–output pairs.

The deep learning system consists of two main processes: offline and online. The system is trained in the offline section to build a complete model. The online process further utilizes this model to predict outputs. In this paper, the instance segmentation network is used for image processing, in which networks that show “perception” and “recognition” eventually give the system the ability to understand the environment and make inferences, thus allowing the manipulator to take appropriate action. In this study, due to the limited amount of data and hardware, we proposed using transfer learning [38], which is a machine learning technique in which a model developed for a task is reused as the starting point for a new model for a different task.

### 3.2. Materials and Methods

This section describes the function of the data argument module used to create training and additional data and focuses specifically on the image processing part used to recognize the target object in each scene.

#### 3.2.1. Dataset

**Figure 2:**
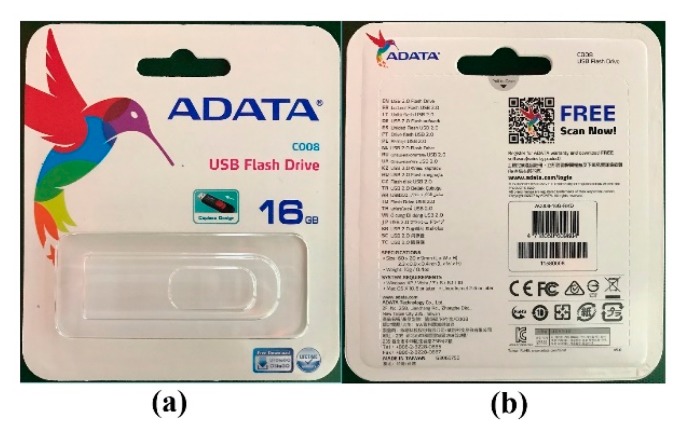
Both front and back sides of the object.

**Figure 3:**
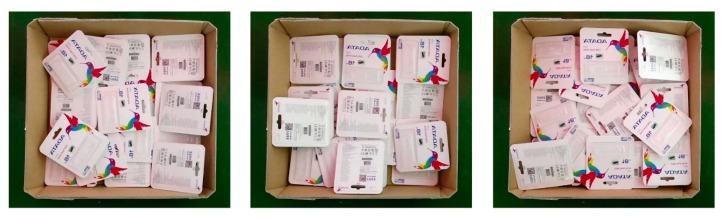
Three examples of actual scenarios.

One popular type of USB flash drive pack served as the target object of this research. Figure 2 shows both the front and back sides of the target object. The situation we hypothesized was similar to actual situations encountered in which there is only one type of object in the scene and the environment is random. Figure 3 presents the three different cases, all of which are similar to cases occurring in reality.

The first step in the procedure was to create the training section dataset. In 2014, Microsoft created a dataset called COCO [39] to help advance research on object recognition and scene understanding. COCO is one of the first large datasets to annotate objects with more than just square or rectangular boundary boxes, making it a useful benchmark to use when testing new object recognition models. Since then, the COCO format has been used to store annotations and has become standard. We therefore converted our dataset to follow this standard so that future research can easily replicate our work. We now introduce the proposed method of data augmentation, which aims to create training data and enrich data under the control of a small dataset without creating noise.

**Figure 4:**
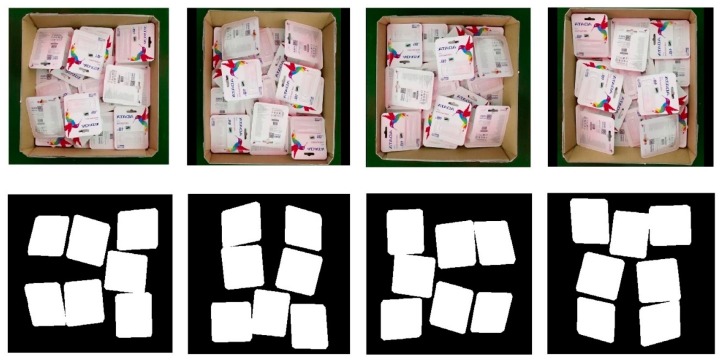
**Top**: Original image and three other versions after the original data passed through the augmentation module. **Bottom**: Corresponding binary image.

To generate the dataset properly with the correct format, the first step was to write a Python script that would annotate the object inside the image. Although each object in the image created an annotated binary image, these were combined to show the similarities between the annotated image and the original image. Once all data were annotated, another Python script was used to handle all annotation formatting details and convert our data into a JavaScript Object Notation format. This kept all image IDs, category IDs, bounding boxes, areas, and image segmentation information in image pixel coordinates. A total of 153 real scenarios were considered and subsequently divided by the ratio 6:2:2 for training, validation, and testing, respectively. In consideration of the limitations of the dataset and to ensure reliable performance, sampling cross-validation was used as *k*-fold stratified random sampling with *k* = 5. The average performance was considered later. To enrich this dataset, the data augmentation module was added to make the training and validation set four times larger than the original dataset. Figure 4 shows the original image and three other versions after they had passed through the augmentation module.

With this amount of data, readers can refer to Table 2 to see how many objects were extracted and annotated based on the original images. The aim of this research was to help solve practical problems in the industry: therefore, the network must learn to detect the best OOIs in the scene. For this reason, the augmentation module was designed to enrich the data source but does not generate instances for both original images and annotated images without a full object in the scene.

#### 3.2.2. Deep Learning Networks

CNNs (convolutional neural networks) have been the gold standard for image classification ever since Alex Krizhevsky, Geoff Hinton, and Ilya Sutskever won the ImageNet challenge in 2012. CNNs have now been improved to the point where they can outperform human beings on the ImageNet challenge [40].

**Figure 5:**
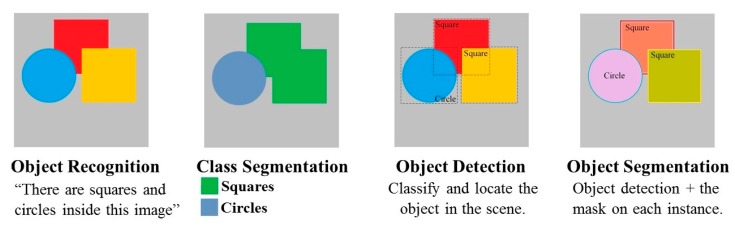
Typical image processing task.

Although these results are impressive, the classification of images is much simpler than the complexity and diversity of true human visual understanding. Thus, the goal is to improve CNNs so that they not only conduct classification but also localize and create the mask for each instance. When exploring computer vision, learners may come across highly similar terms such as “object recognition”, “class segmentation”, and “object detection”. This may be confusing at first; however, observing the functions that it can perform provides clarity. Figure 5 presents examples of the output information that the network can provide, with increasing task difficulty from left to right. The most difficult task, defined as “object instance segmentation”, is for the network to automatically label all the shapes in an image and identify their location down to the pixel level. In this study, our approach was to select and use the most advantageous network that could perform “object instance segmentation” and output a high-quality segmentation mask for each instance in a heavily cluttered environment.

He et al. [41] presented their work as Mask R-CNN, which is considered state-of-the-art in instance segmentation. With some modifications, this was used in the visual perception module to realize the CNN-based semantic segmentation function to fit our problem. The capabilities of Mask R-CNN developed from the initial use of the CNN to detect objects in an early application known as R-CNN [42]. Later, Fast R-CNN [43] and Faster R-CNN [44] were developed to lift the ability of the neural network to a new level. From R-CNN to Faster R-CNN, CNNs play an important role in effectively locating different objects in an image with bounding boxes. Beyond this, Kaiming He and other researchers, including Girshick, expanded the techniques used in Faster R-CNN to steps that could locate the exact pixels of each object instead of bounding boxes. This was the aforementioned Mask R-CNN, the main foundation of our visual perception module. The only difference between Mask R-CNN and Faster R-CNN is that Mask R-CNN was expanded by adding a branch to predict an object mask in parallel with the existing bounding box recognition branch. This difference led to changes in the objective function: moreover, this object mask has been found to be critical in obtaining good results [45]. Mask R-CNN is also a two-stage deep object detector inherited from Faster R-CNN.

**Figure 6:**
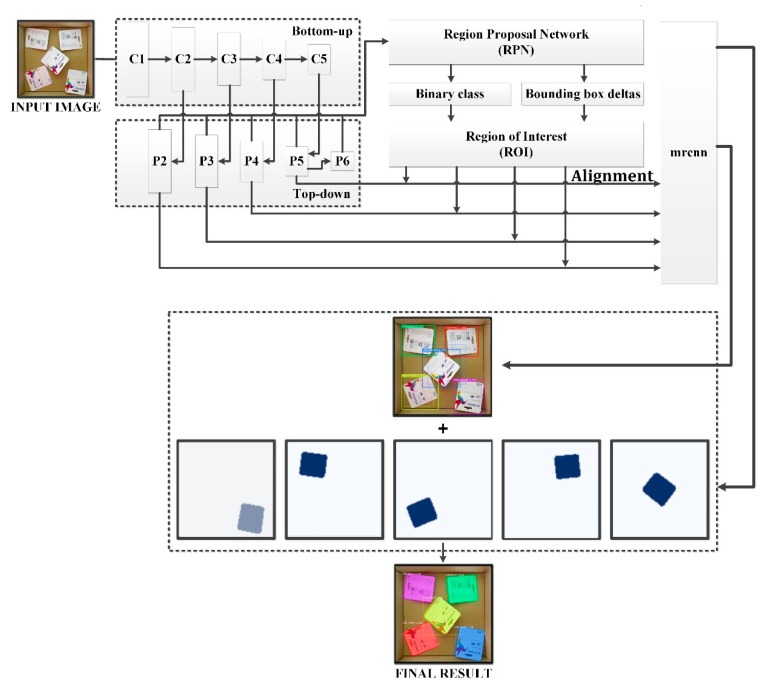
Implementation of deep learning for image processing.

Figure 6 illustrates the overall procedure and implementation of Mask R-CNN architecture in the current study. In the first state, known as a region proposal network (RPN), candidate bounding boxes within the input image are proposed. A small model within the overall network is responsible for proposing the bounding box candidate and needs to be trained by optimizing the parameters to minimize the cost function. Whenever the network contains these areas, a series of subsequent steps helps the system to select the best areas with the highest likelihood of containing the object while refining the candidate’s boundary boxes to fit the object as much as possible. These selected areas are used as the input for the second stage. The system once again calculates the overlap between the proposal areas and the bounding box of ground truth. At this point, the system requires a metric to evaluate how good the overlap values are in general cases, which are independent of the size of the objects in pixel coordinates. Intersection over union (IoU) [46] is a standard metric that can be used in such a case. The IoU metric was used to evaluate the accuracy of the proposed visual perception module. The region of interest (RoI) areas, which have the highest IoU values and the lowest values to be selected with a fixed ratio between them, are the input for the next step. The network ignores the neutral values. Based on the IoU values, the network easily separates the selected RoI into two groups: positive RoI and negative RoI. Target class IDs, bounding box deltas, and masks are then selected based on the RoI threshold of 0.5. Together with the output from the previous step, these RoIs are used to train another network containing two graphs that output the final predicted class, bounding box refinement, and masks for each instance. However, RoiPool, which is not designed for pixel-to-pixel alignment between network inputs and outputs, can only perform coarse spatial quantization for extraction of features. It was therefore replaced by RoiAlign. Optimizing the final three predictions corresponding to the ground truth helped to improve the accuracy of the network during the training time. Based on the number of classes in the dataset and its size, a fixed number of epochs and steps per epoch were set before the training was defined. The network converged after the fixed number of epochs, and the best model was selected as the final model for the inference mode.

## 4. Appropriate 3D Pose Estimation

**Figure 7:**
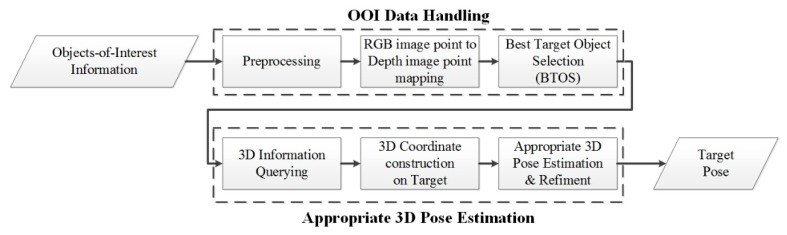
Flowchart of the proposed 3D object pose prediction method.

In this section, we describe in detail how to predict the appropriate 3D pose for picking up the target in the scene. The results were later used to control an industrial robot arm responsible for picking the object. The output from the visual perception that contains information regarding all of the best OOIs in the scene was used as the input for this module. Figure 7 presents a flowchart of the proposed 3D pose estimation, which comprises two main parts: OOI-DH and 3D pose estimation. Data preprocessing is performed in the first step of OOI-DH and ultimately returns the center points of the objects. The next step is to map point to image depth coordinates from RGB image coordinates. These values are used to construct a 3D point, thereby allowing the system to determine the relative distance of the object from the camera.

**Figure 8:**
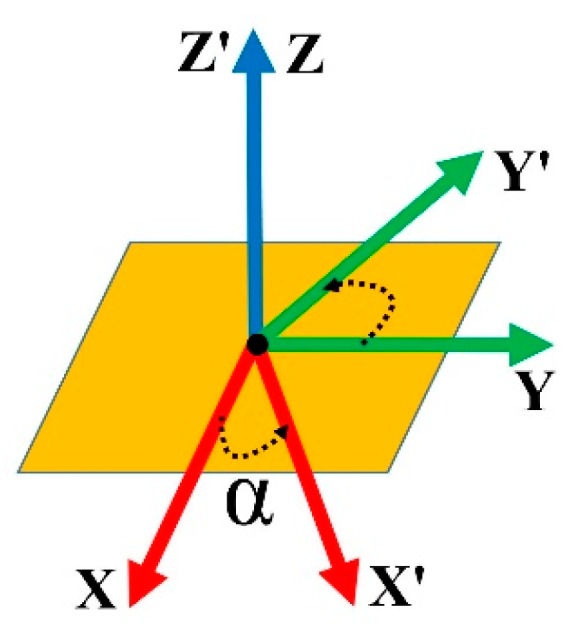
Example of two acceptable coordinate systems on the target plane.

The next step comprises processing to select the final target and collecting the required information on this target before estimating the appropriate 3D pose for picking the target object. Since the translation values can be reused from the previous step, only an appropriate coordinate system needs to be built. The system first collects sufficient points that are relatively close to the target point in 2D before mapping to the 3D environment. Based on the 3D information, a predicted plane is constructed using the plane segmentation method. Finally, an appropriate coordinate system is built based on the built plane. We enhanced the accuracy of the final translation results by using a linear regression model as a refinement method. It is sufficient to only determine the normal vector for the highest contact probability of the suction pad to pick up the object at the target point on the object’s surface. Figure 8 describes an example of two possible coordinate systems on the target plane where the *x’–y’–z’* coordinate system is created by rotating the *x–y–z* coordinate system along the *z* axis at an α angle (α > 0). For picking up the object, both the coordinate systems are acceptable for calculating the final pose. A simple technique is used to simply define an appropriate rotation matrix. All translation and rotation information is transformed corresponding to the robot base’s coordination. At this stage, the program is able to send execution commands to the robot to pick up objects.

### 4.1. OOI Data Handling

Based on the results of the deep learning network, the system can easily determine the total number of instances in the prediction. It then needs to know exactly how many classes there are in the scene before separating this information into the same group if it belongs to the same class. The network only needs to retain the best results: therefore, the next step is to remove the bad objects and retain good objects that have a mask area that is higher than the lower threshold but lower than the upper threshold. Another filter is then set up to remove all components that have a confidence score lower than a fixed threshold before the system calculates the center of the remaining object.

**Figure 9:**
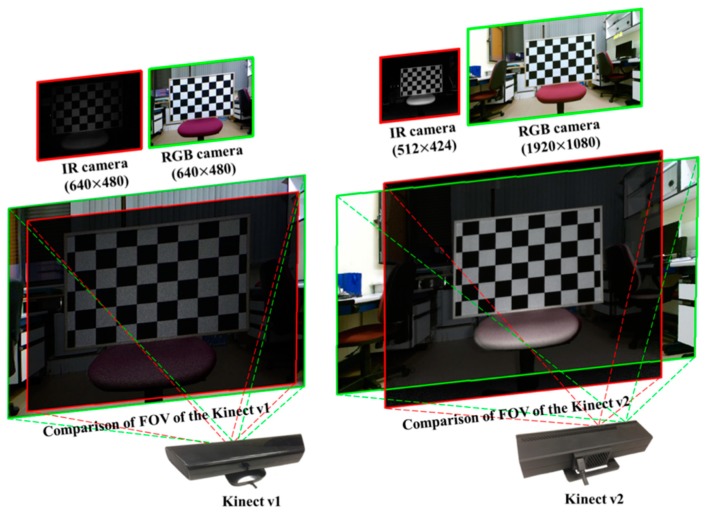
Kinect v1 and v2 overlap regions in the captured scene. The green rectangle represents the RGB view, and the red rectangle represents the IR view [47].

**Figure 10:**
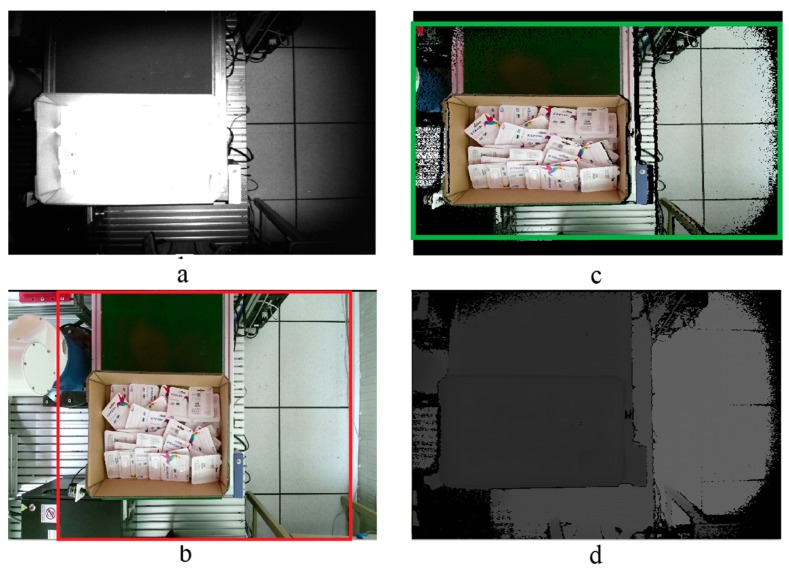
(**a**) IR image, (**b**) original image, (**c**) registered color image and IR obtained from transformation and cropping, (**d**) depth image.

Having obtained the object center information in the RGB color image coordinate, the next step is to find corresponding points in the depth image coordinates so that the system can later query the 3D information. As mentioned, Kinect v2 was selected as the 3D camera in this study. A recent study compared both versions of Kinect in terms of the RGB and IR FOV, which is not mentioned in the official product specifications [47]. In Figure 9, the overlap between two types of Kinects in relation to the RGB view and IR view is presented. The difference between color image resolution and depth image resolution indicates that the calibration methods designed for Kinect v1 cannot be applied to Kinect v2. This is a common problem; however, very few studies have mentioned this issue, and we found only one study that focused on solving this problem [48]. In the aforementioned study, the authors corrected the radial distortion of the RGB camera and determined the transformation matrix for the correspondence between the RGB image and the Kinect v2 depth image. However, a rigorous analysis of the projection matrix in this study revealed that it is correct in certain cases and cannot be generalized when *x* ∈ [0, 1920] and *y* ∈ [0, 1080], where *x* and *y* denote the pixel values in the RGB color image coordinate. To overcome this limitation, we first verified the effect of the differences between the RGB view and the IR view. Figure 10 shows the results of an experiment in which we used libfreenect2 library on Kinect v2. The bottom-left image is the raw RGB color image, and the top-right image presents the results after registration and cropping (RGB with depth). Clearly, the RGB with depth images is the intersection between the raw RGB colors and IR images. Because the FOV is different between the color image and IR image, the color image is cropped on both sides to fit the FOV of the IR image. Readers can verify this easily by looking at Figure 10b,c. Only the information inside the red box in the RGB color image appears inside the RGB image, with depth in the green box area. The upper and lower part of the RGB with depth is black, which means that there are no data because the FOV of the color image is smaller than the FOV of the IR image. After the calibration and cropping process, the real size of the RGB with a depth image was changed to 512 × 360 [48]. The original size of the color image was 1920 × 1080 and that of the IR image was 512 × 424. Because the FOV was different, we determined that the bottleneck of our entire system was a method for mapping from a point in the color image to the point that has the same relative position in the depth image. A new method was proposed to find the correspondence between the RGB and depth image of the Kinect v2 to solve this bottleneck.

**Figure 11:**
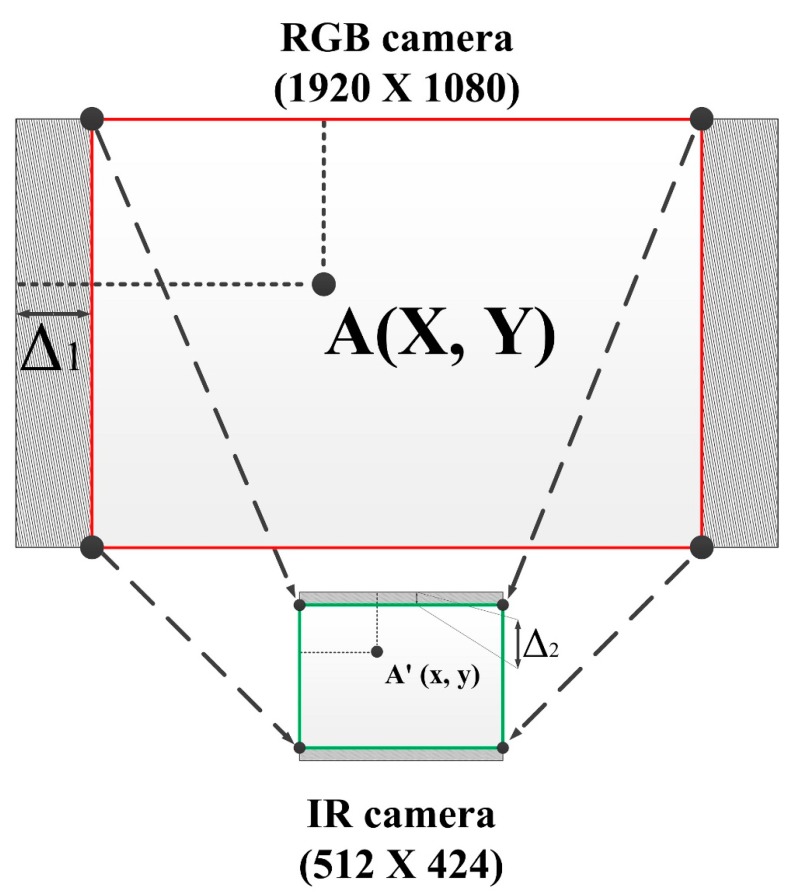
Demonstration of mapping methodology for Kinect v2.

Figure 11 will make it easier to explain the mapping process. The red area represents the overlapped area between the FOV of the RGB camera and the FOV of the IR view. The color image is cut on the left and right side, while the height remains the same at 1080. The scaling ratio must be the same for both height and width. From all of these arguments, it is easy to figure out the scaling value:

(1) $$k=\;\frac{h_{1}}{h_{2}}=\;\frac{1080}{360}=3,$$

where *k* denotes for the scaling value, $h_{1}$ denotes the height of the overlap view between the RGB and IR view (before scaling), and $h_{2}$ denotes the height of the RGB with depth (after scaling).

Suppose that the point *A* ($x_{1}$, $y_{1}$) in the RGB image coordinates corresponds to the point *A*’ ($x_{2}$, $y_{2}$) in the depth image. From Equation (2), the mapping function between the RGB color view and the IR view (depth image) presents as

(2) $$x_{2}=\;\frac{x_{1}-\;{∆}_{1}}{3}\;y_{2}=\;\frac{y_{1}}{3}+{∆}_{2}.$$

As mentioned, a linear regression model was used to improve the mapping results. The ${∆}_{1}$ and ${∆}_{2}$ are affected by the values of $x_{1}$ and $y_{1}$ in the RGB color pixel coordinate. Thus, a model to predict the offset pixel values of ${∆}_{1}$ and ${∆}_{2}$ was built using a sufficient amount of data as the input for training. The final results are shown in Equation (3) below:

(3) $${∆}_{1}\;=\;-0.02814\;\times \;x_{1}\;-\;0.00704\;\times \;y_{1}\;+\;298.656\;{∆}_{2}\;=\;-0.00190\;\times \;x_{1}\;+\;0.00971\;\times \;y_{1}\;+\;26.427.$$

**Figure 12:**
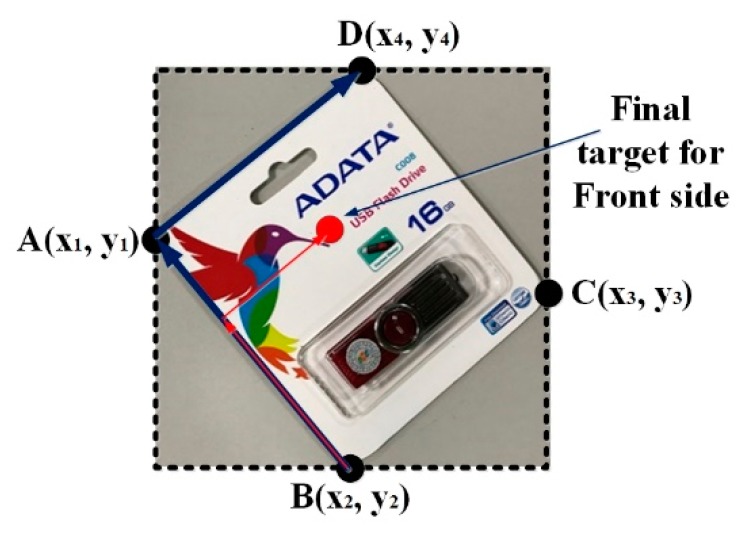
Method of calculating the final target for the front side case.

The mapping values in the depth image coordinate form the basis for the BTOH module before the system starts to estimate the 3D pose of the object with respect to the coordinate of the camera. The first step taken by the BTOH is to extract the 3D information using the libfreenect2 library [49], an open source for Kinect v2. Based on the depth value of each object, the system keeps the one with the shortest distance as the only target. In our study, the reference point for the back side of the object was in the middle. However, for the front, the target point in an asymmetrical image is the point with a fixed scale. To address this problem, we used a technique called feature matching and then found the perspective transformation to figure out the four corners of the objects, shown as points A, B, C, and D in Figure 12. An image called a query image containing the object of interest is prepared to use the feature matching technique. The algorithm identifies the features inside the query image and the best matches of these features inside the target image. Two sets of points from both images are used to find the perspective transformation of the query image in the target one. In our setting, the four corners of the query image match the four corners of the object. From this, the orientation of the object in a 2D image coordinate can be observed. Finally, with a fixed ratio as a constant that can be calculated on the basis of the predefined position on the target object, we determined the final target position based on basic geometry and vector knowledge. For the final target in the RGB color coordinate, the system only maps and acquires 3D information (*x*, *y*, *z*) for the front side case. This procedure does not need to be repeated for the back side.

**Figure 13:**
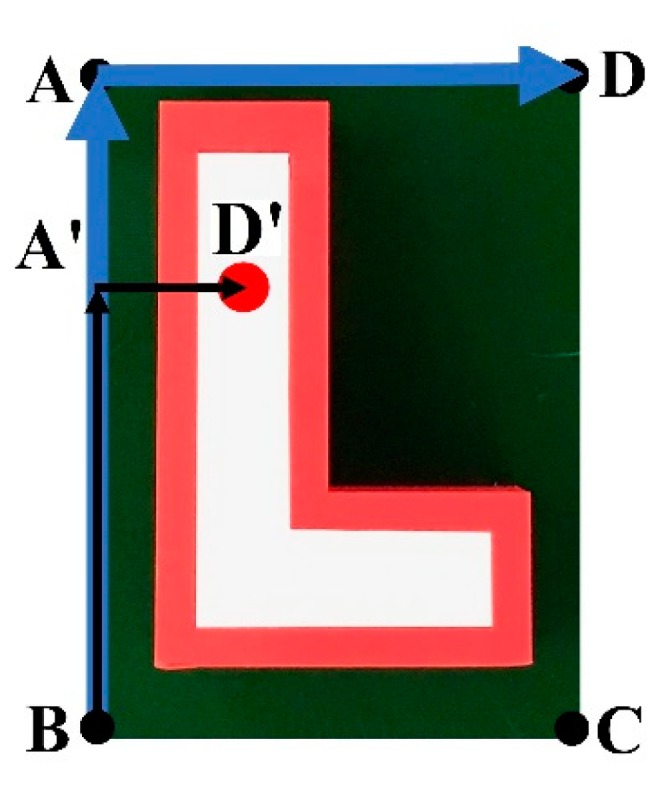
Example of the nonrectangular object with background used as the query image with reference vectors to locate the destination point.

**Figure 14:**
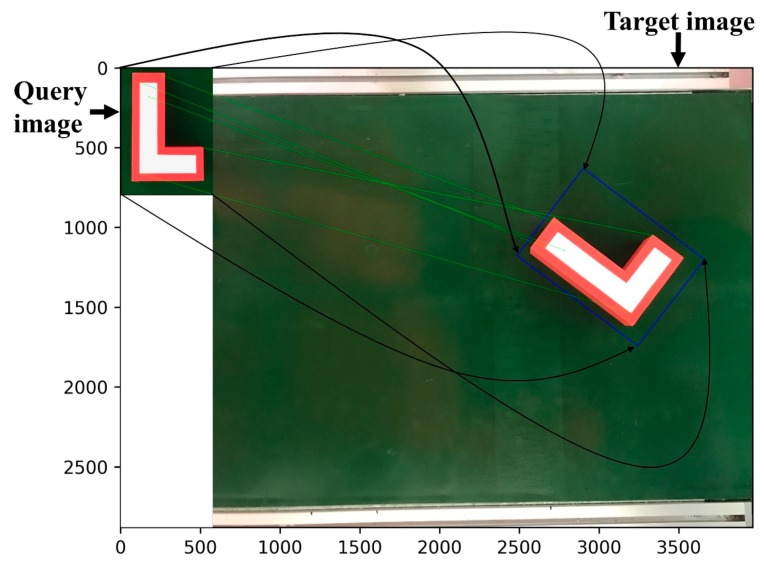
Overall results of the proposed technique on a nonrectangular object.

This technique also works for objects with a nonrectangular shape under a proper set-up. Figure 13 shows an example of a query image in an “L shape”, with the red color point being the target position. Any destination point in the query image can be represented using vectors with fixed modules and directions starting from one of the four corner points (A or B or C or D). To represent the target point in Figure 13, the two reference vectors ($\vec{BA}$ and $\vec{AD}$) are first created from the original three corner points of the query image. Based on the reference vectors, $\vec{BA^{\prime }}$ and $\vec{A^{\prime }D^{\prime }}$ are identified. The ratios between the modules of $\vec{BA^{\prime }}$ and $\vec{BA}$ and $\vec{A^{\prime }D^{\prime }}$ and $\vec{AD}$ are calculated for further location of the target points in the target image. Figure 14 shows the overall results of the proposed technique. The small image in the upper-left corner is the query image, while the large image is the target. The technique exactly matched some of the features in the query image to those of the target one, as shown by the green lines. These features in the target image in combination with the relative positions (as illustrated by the blue box) of the four corners of the query image help to identify the final target.

### 4.2. The Appropriate 3D Pose Estimation

To conduct the appropriate 3D pose estimation for picking up the objects, our approach was to find a method for building a proper coordinate system on the predicted plane. Because we had already obtained the translation values of the target point in the first step, the task was to create a rotation matrix at that point with respect to the coordinate of the camera. The linear regression model was used to refine this translation result, which later demonstrated impressive accuracy.

The depth error of the two versions of the Kinect sensors is described as a function of the distance between the device and the object [50]. Here, the *x* coordinate and *y* coordinate corresponding to the image coordinates of a pixel are calculated based on the *z* coordinate [51]. Therefore, the final translation values are related to the image coordinates of pixel values. In this research, a linear regression model is used directly on the output of OOI-DH as a refinement technique. The final translation prediction results ($x_{pr}$, $y_{pr}$, $z_{pr}$) in millimeters are shown in Equation (4) below:

(4) $$x_{pr}=\;0.98966\;\times \;x\;-\;0.01493\;\times \;y\;-\;0.04134\;\times \;z\;+\;12.07\;y_{pr}=0.02155\;\times \;x\;+\;0.99117\;\times \;y\;+\;0.02561\;\times \;z\;+\;16.69.\;z_{pr}=0.02105\;\times \;x\;+\;0.01863\;\times \;y\;+\;0.90290\;\times \;z\;+\;5.30$$

**Figure 15:**
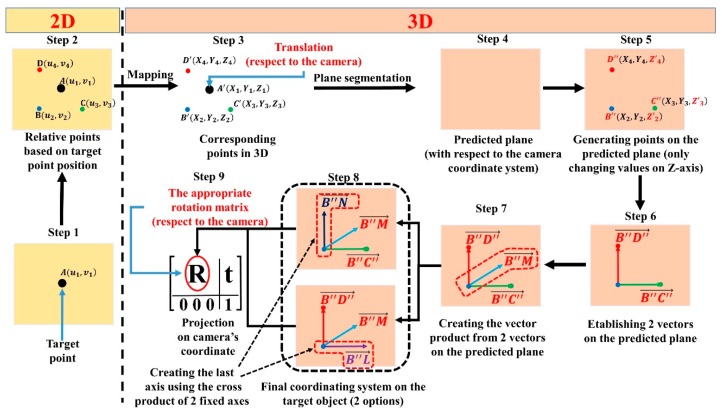
Procedure for building an appropriate coordinate system on the target object to create a rotation matrix with respect to the camera.

To predict a rotation matrix for picking up objects, the system first builds a predicted plane using plane segmentation [52,53]. After that, an appropriate coordination system is built on that predicted plane. Figure 15 shows the whole procedure step by step as follows:

- **Step 1.** Determine the target point in the 2D RGB image. This step is done by OOI-DH module;
- **Step 2.** Collect sufficient relative points based on the target point in the 2D RGB image and choose the three key points, i.e., B, C, and D. B, C, and D are used to build the appropriate coordination system;
- **Step 3.** Map and acquire 3D information from the three sample points (B, C, and D) to create B’, C’, and D’, respectively;
- **Step 4.** Create the predicted plane in 3D based on B’, C’, and D’ using the plane segmentation method;
- **Step 5.** Use B’, C’, and D’ to create the three new respective points B’’, C’’, and D’’ on the predicted plane by finding new *z* values while keeping the *x* coordinate and *y* coordinate unchanged;
- **Step 6.** Create two vectors, $\vec{B^{\prime \prime }C^{\prime \prime }}$ and $\vec{B^{\prime \prime }D^{\prime \prime }}$;
- **Step 7.** Generate a vector product $\vec{B^{\prime \prime }M}$ from $\vec{B^{\prime \prime }C^{\prime \prime }}$ and $\vec{B^{\prime \prime }D^{\prime \prime }}$. The direction of the vector product must be considered for the robot motion to pick up objects;
- **Step 8.** Generate an additional vector product either using $\vec{B^{\prime \prime }M}$ and $\vec{B^{\prime \prime }C^{\prime \prime }}$ or $\vec{B^{\prime \prime }M}$ and $\vec{B^{\prime \prime }D^{\prime \prime }}$ to create a coordination system. The output system of this step can be either the Cartesian coordinate system ($NC^{\prime \prime }M)$ or ($D^{\prime \prime }LM)$;
- **Step 9.** Project the built coordination system on the camera coordination system to define the rotation matrix.

Finally, precise information on the pose of the object with respect to the camera’s coordinate is obtained. This transformation of the object to the camera frame is presented as the homogeneous matrix TOC∈SE(3).

Another transformation, TCR∈SE(3), which specifies the relative position and rotation of the camera with respect to the robot pose, remains constant when the relative position of the camera is fixed with the industrial manipulator. This camera-to-robot transformation can be obtained by performing a calibration procedure known as hand–eye calibration, as described in References [54,55,56,57].

**Figure 16:**
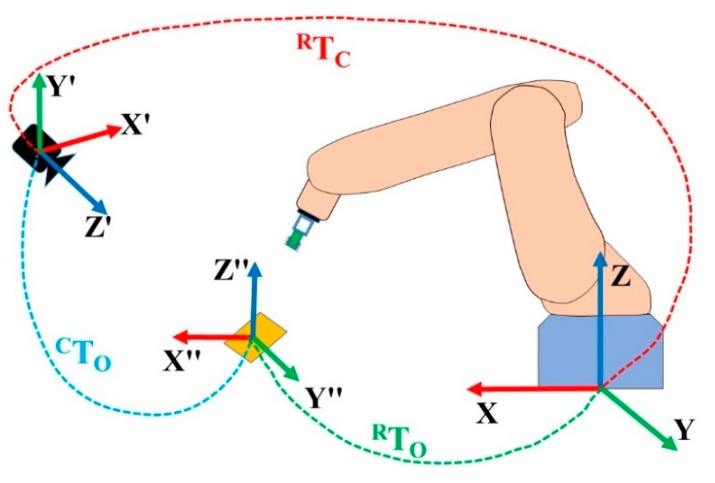
Camera and object position with respect to the robot base.

Figure 16 shows all the relationships and matrix of the corresponding transformation. Once all of the relationships have been established, the final target, camera-to-robot transformation TOR∈SE(3), can be calculated using Equation (5):

(5) TOR=TCRTOC.

However, readers are recommended to follow the method [58] for obtaining a simple solution by computing Euler angles from a rotation matrix for robot operation.

### 4.3. Performance Evaluation of the Appropriate 3D Pose Estimation

**Figure 17:**
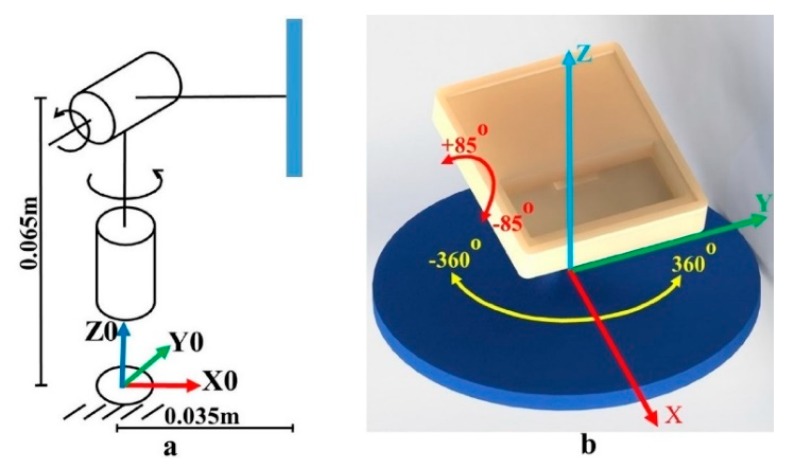
(**a**) Kinematic model, (**b**) a supportive platform to capture the object’s ground truth with respect to the robot (for both the front and back sides).

The ground truth of the object with respect to the robot base is one of the most important elements used to evaluate the accuracy of the 3D pose estimation module. It is challenging to observe the absolute pose of the objects. We used an indirect way of obtaining the ground truth of a planar object in the 3D environment on our own target object. Since we start from the beginning, the proposed method was chosen to return the pose of the object with respect to the robot’s base. This way can help to achieve a more objective result by eliminating the errors that are hard to measure during the process to obtain the ground truth using the transformation from the camera to the robot’s base. This indirect way was used to show that the pose error attained with our method is small enough for a bin-picking system. Figure 17 illustrates the proposed platform to obtain the ground truth information. The original coordination of this platform, located at the center of the base, provides translation values and appropriate rotation information with respect to the robot base.

The platform can rotate 360 degrees in the *x*–*y* plane, and the upper part can rotate from −85° to 85° in the *z*–*x* plane. Based on this design, the ground truth with respect to the robot is identified using the end effector as a tool to define the relative position between the base of the platform and the robot base. Because the equation can be established using forward kinematics equations, it is easy to calculate the respective six poses at the point on the plane where the target object is placed. Equation (6) shows the translation and rotation estimation errors of the proposed method, which are determined using a basic and effective method for measuring estimated errors:

(6) $$\delta T_{A}=T_{A}-\;{\hat{T}}_{A}\wedge \delta R_{A}=R_{A}-{\hat{R}}_{A}.$$

In Equation (6), *A* = {x,y,z} denotes one of the three axes of the 3D Cartesian coordinate system. $T_{A}$ and $R_{A}$ represent the ground truth of the object’s translation and rotation, and ${\hat{T}}_{A}$ and ${\hat{R}}_{A}$ represent the prediction of translation and rotation, respectively. Performance is evaluated on the basis of the mean absolute error (MAE) metric used by Lin et al. [4], as presented in Equation (7):

(7) $$\mathrm{MAE}(\delta E_{A})=\frac{1}{N}\sum \left|\delta E_{A}\right|,$$

where *N* represents the total test number and E= {T, R} denotes either the translation or rotation variable.

## 5. Experimental Results and Discussion

### 5.1. Image Processing Results

The network can fully train from scratch using a large number of datasets and strong hardware with a good minibatch image. Because we had a small dataset and mediocre hardware performance, we used the pretrained model. This model provided the initial values for all of the weights within the network, which was trained on the large COCO dataset in a synchronized 8-GPU implementation (0.72 s per 16-image minibatch) [41].

**Figure 18:**
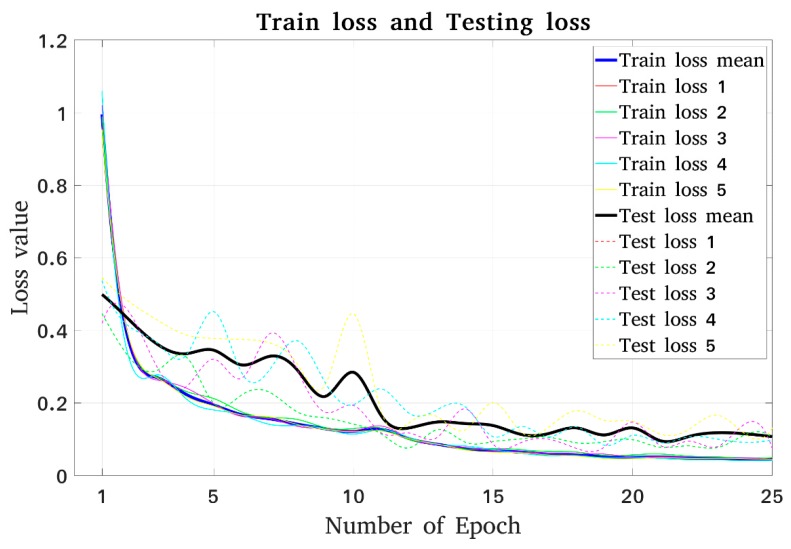
Loss graph for the deep learning model.

Our model was trained on a single GTX1080Ti with only one image per GPU. The training was divided into two small parts with a total of 25 epochs. In the first 10 epochs, RPNs, the first step in the overall training process, were trained separately with an initial learning rate of 0.001 and without the use of MS COCO pretrained weights. At this stage, all of the layers of the backbone were frozen, and only the randomly initialized layers were trained. Because this was a small network and our own objects did not appear in the COCO dataset in the 80 total classes, we needed to retrain our model. Using pretrained weights as initialized values with the same learning rates, we trained all layers of the networks in subsequent stages after finishing training on the RPN. The training stopped after the 25th epoch had finished. Figure 18, which provides basic information regarding the learning performance of the overall process, shows the loss graph for the training and validation process. This shows that the training loss decreased very rapidly in the first few epochs and continued to decrease until the last epoch. During this training time, the amount of loss in the validation set also decreased and almost converged after the 12th epoch until the training finished with a small fluctuation. After five runs, it was clear that there was no significant variation in training and testing performance. The difference between training and testing was almost constant, and the models all converged at epoch = 25. Thus, increasing the number of epochs led to an overfitting problem. This problem could be expressed as the training loss continuing to decrease while the testing loss began to increase with strong variation.

**Figure 19:**
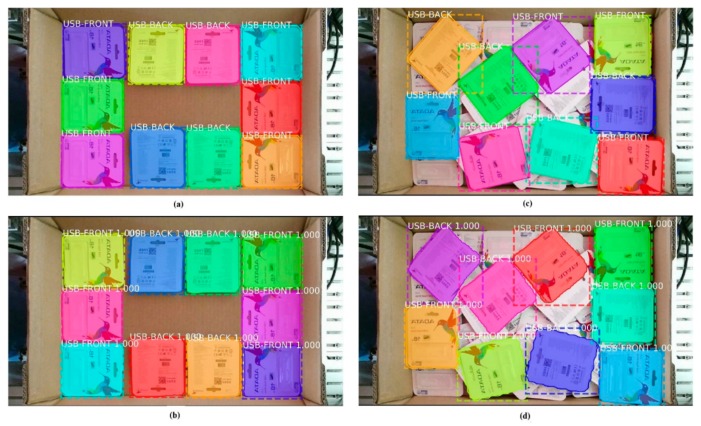
(**a**,**c**) Ground truth of two test cases; (**b**,**d**) the results of the prediction corresponded to two ground truths.

**Figure 20:**
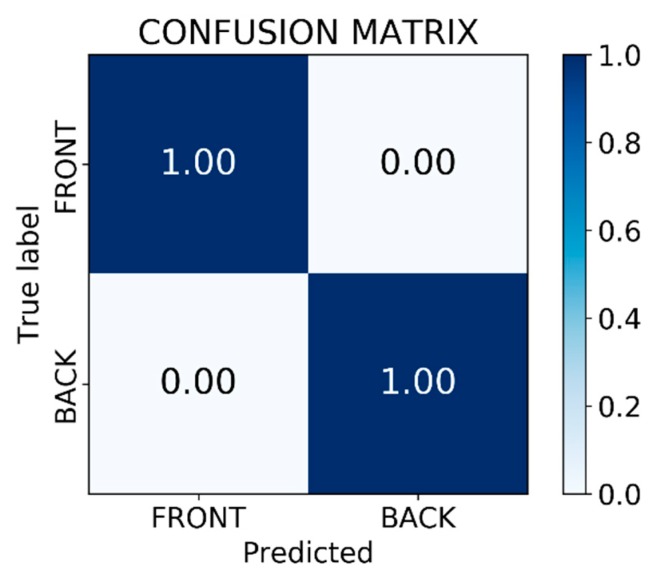
Confusion matrix of the classification results from the model.

**Figure 21:**
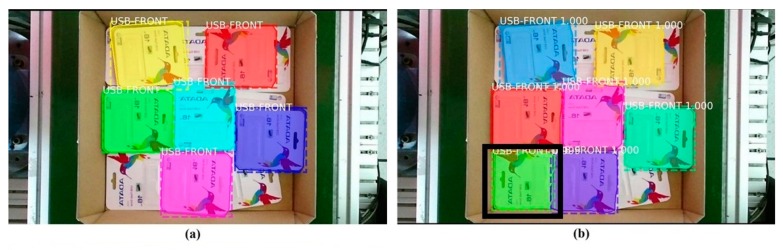
Another case in the test set—(**a**) ground truth; (**b**) the results of the prediction.

The model was then used in inference mode to run on the test dataset to check the accuracy of the model on the unseen data. Figure 19a,c shows the ground truth of two cases, and the results of correspondence detection are shown in Figure 19b,d, respectively. As shown in Figure 20, the normalized confusion matrix further showed the model’s classification accuracy on the test set. The result indicates that the model could successfully classify all objects. Figure 21 shows another successful prediction and creation of the mask for each instance. The final prediction gave us six instances, whereas the ground truth had only five instances, because we forced the network to only detect the full objects. The network still gave us the correct classification, and the final prediction for that instance was the front side of the object marked in the black box. The system could then be improved by using filters to remove less accurate predictions.

In this study, both the accuracy of classification and locating the object’s position in the image strongly affected the final result. For an overall assessment, the model was used to run the test set, the results of which are shown in Table 3. It is evident that with typical IoU thresholds of 50% and 75%, identical results were obtained over five runs with 100% accuracy. In addition, the mean average accuracy with a threshold value from 0.5 to 0.95 at step size 0.05 demonstrated that the overall accuracy was 91.18%. This surpassed the state-of-the-art segmentation method. These reliable results provide a firm basis for implementing the next steps of the proposed method.

### 5.2. The Appropriate Pose Estimation Performance

**Figure 22:**
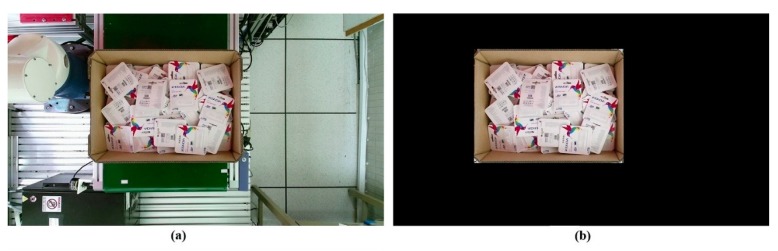
Image preprocessing to remove unnecessary information—(**a**) the original RGB image captured by the camera; (**b**) the result after removing the unnecessary information.

To make the entire process easy to follow, the real implementation is shown from the beginning. The process began with capturing the image using Kinect v2 as the first step in the inference mode. Figure 22a is an image captured by Kinect v2 that was later subjected to preprocessing to remove all unnecessary information, as shown in Figure 22b. Although the results in the test set during the training process indicated that the model functioned extremely well, the results from the deep learning network were raw results that needed to be processed to achieve the highest efficiency.

**Figure 23:**
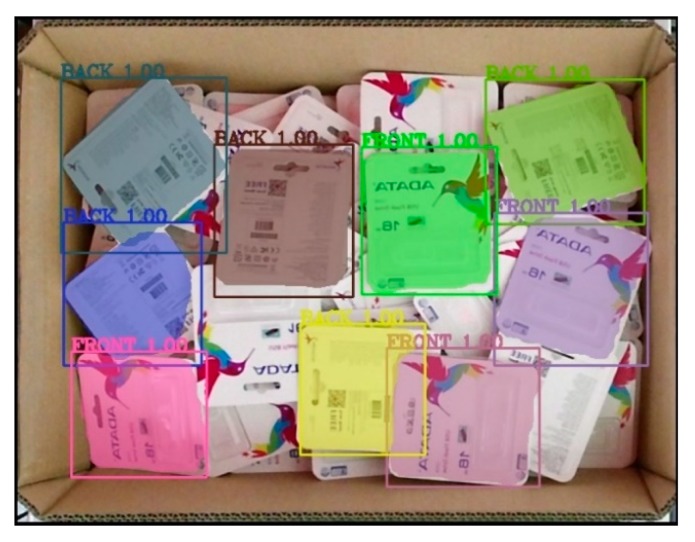
Raw output from the deep learning network.

**Figure 24:**
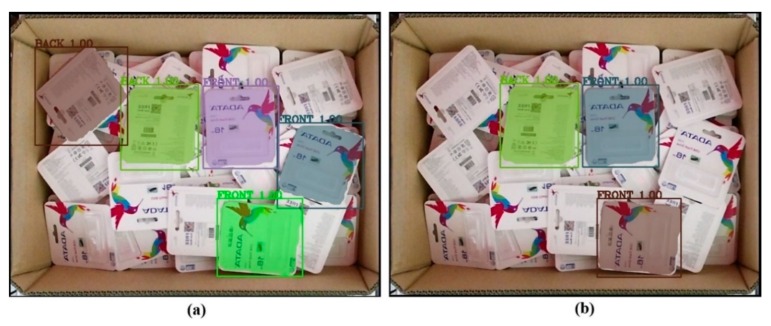
Result after passing through the filter at a fixed area threshold percentage criteria: (**a**) 90%, (**b**) 95%.

Figure 23 presents raw output from the deep learning network. The network still detected the object with partial object occlusions. To tackle this problem, we calculated the mask area by counting the number of pixels belonging to one mask before dividing by the reference value what was supposed to be the value if it were a full object for calculating the percentage. Later, the system removed the output with a percentage value less than a predefined threshold. In the following discussion, the predefined threshold was set to be either 90% or 95%. Figure 24 further illustrates the results after removing unexpected outputs below the two aforementioned thresholds. In Figure 24a, five objects were retained for the following stages, whereas the results in Figure 24b retained only three objects.

**Figure 25:**
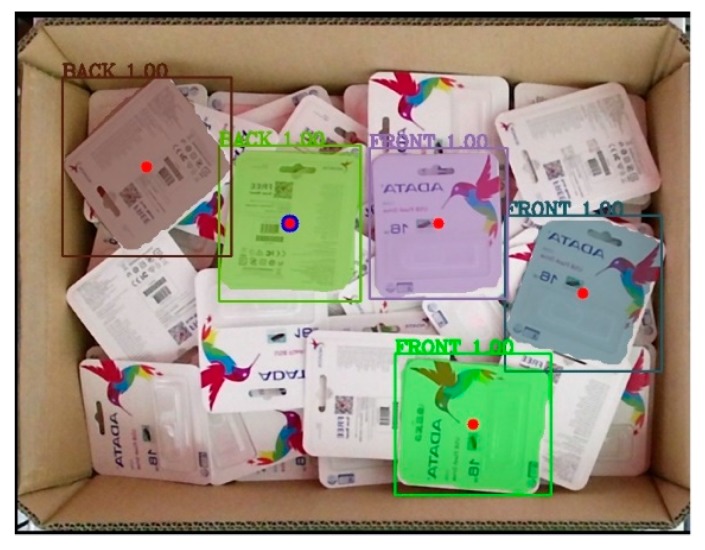
Result after calculating the center points (red points) and deciding the final target (blue point).

Following this stage, each center point was calculated on the basis of the object’s bounding box. The five red points in Figure 25 denote the center points of the objects. In this case, the object with the larger blue point was the final target after considering the relative distance with respect to the camera. This result corresponded with Figure 24a.

**Figure 26:**
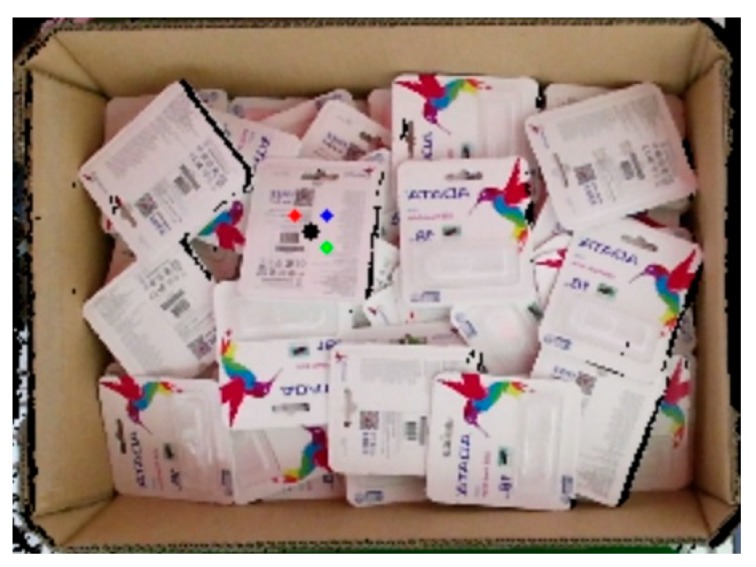
Result of mapping from a point in the color image to a point that had the same relative position in the depth image.

**Figure 27:**
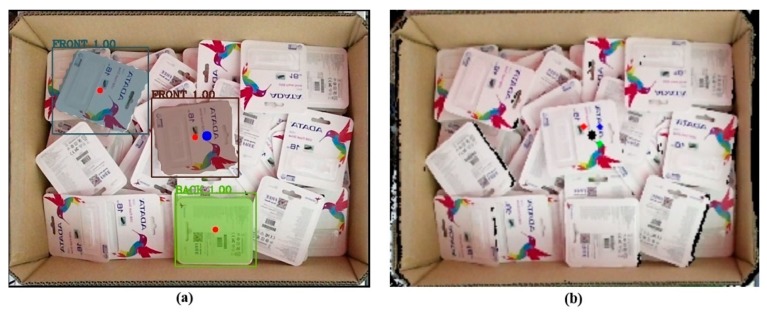
(**a**) Final target as the blue point, and (**b**) mapping result as the black point.

The next step was to map from the final target point on the RGB color image coordinate to the RGB with a depth image. The black point in the RGB with a depth image, shown in Figure 26, was the result of blue point mapping, as displayed in Figure 25. With normal vision, the mapping accuracy was determined to be favorable. The result indicated that the final target in this case was the back side. In Figure 27, another example is presented in which the front side was the final target. The whole procedure in this case followed the method using a feature matching algorithm. Clearly, the mapping method worked in this case. Its performance is evaluated in the following section.

**Figure 28:**
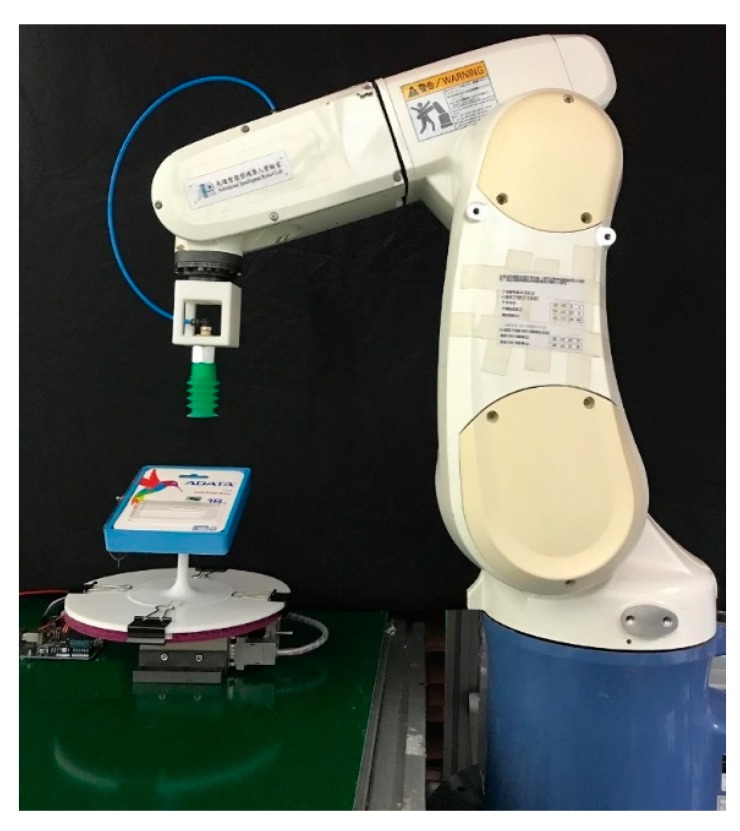
Support platform to take the ground truth of objects in the working area.

To evaluate the accuracy of the proposed 3D pose estimation, the object was placed on the platform at a fixed position with respect to the robot, as shown in Figure 28. The real two degrees of freedom platform was designed to totally fit with both the front and back sides. In line with the original intention, this platform could rotate 360° at the first joint and also rotate on the upper part. It was flexible enough to represent all possible situations for a single object in this scenario. The ground truth was easily calculated because the relative position between the platform and the base of the robot was fixed and observed using the teach pendant. By giving two joint angles of the platform, the relative position and the rotation of the target plane and target point were retrieved as the ground truth of the object with respect to the robot base. In our experiment, the performance was tested on a total of 25 cases for one side. The first joint rotated from 0° to 360° with a step size of 45 (eight cases in total). The second joint rotated 10°, 20°, and 30° relative to the ground surface at each position after the first joint had moved. The final case was a special one in which the second joint was equal to zero, which meant that the target plane was parallel to the ground surface.

The results obtained for both the back and front sides are presented in Table 4 and Table 5, respectively. The mean average errors for all poses are listed in Table 6. In addition, the system demonstrated stable performance with an overall mean translation error of less than 2.3 mm in all directions and a mean rotation error of less than 2.26° on all axes. Lin et al. [4] presented their results on the same target, where average translation and rotation errors in the three axes were less than 5.2 mm and 3.95°, respectively. In comparison, our average error improved by reducing average translation errors and rotation errors by 2.9 mm and 1.69°, respectively. The real dimensions of the object used in the entire system are presented in Table 7. For planar objects, the deviation on the *x* and *y* axes determines whether the system is good enough to perform random object picking tasks, whereas deviation on the *z* axis is less important because the suction pad can easily change within a certain range in the *z* direction. The maximum average percentage error in the result of the translation estimate was 2.15% along the *x* and *y* axes. This level of error ensured that the proposed method worked well on the planar object in the cluttered environment for random bin-picking tasks. These results demonstrated the validity of the proposed system’s pose estimation module.

In light of the above discussion, feature matching plays an important role if the target object is on the front side. Therefore, in the case of feature matching being mandatorily used, the proposed method for building the appropriate pose may not work correctly on planar objects with insufficient or no texture. In the future, to ensure that the system can robustly tackle textureless planar objects, a solution to replace the feature matching technique should be further investigated.

### 5.3. Computational Efficiency and Picking Performance

The entire system ran on an Ubuntu 16.04 platform equipped with an Intel^®^Core^TM^ i7-8700 CPU @ 3.20GHz x 12 (Intel Corporation, Santa Clara, CA, USA), 16-GB DDR4 system memory. Our graphics card was NVIDIA GeForce GTX 1080Ti (NVIDIA Corporation Santa Clara, CA, USA) with an 11-GB frame buffer memory. As indicated in Table 8, the average total processing time was approximately 0.997 s for the front side and 0.727 s for the back side. The deep learning part clearly takes up more than half of the entire processing time: therefore, with higher computational efficiency, the total processing time would be shorter than the value given in this study.

**Figure 29:**
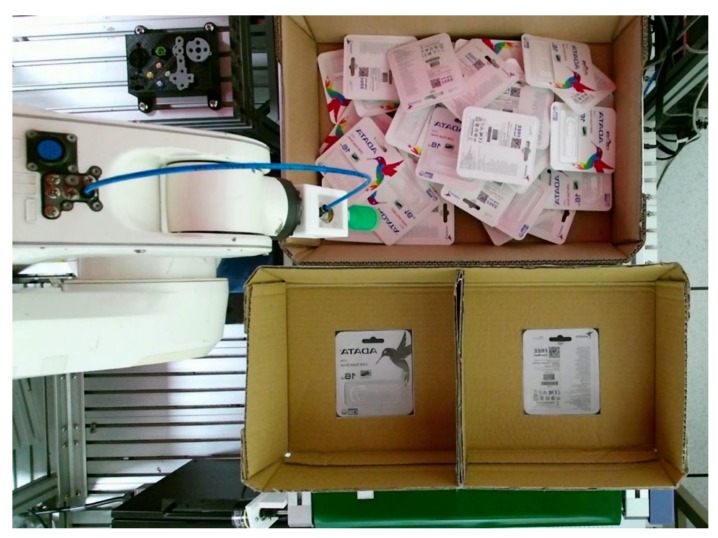
Set-up for evaluating the final pickup success rate.

Finally, the accuracy of the random bin-picking system was verified to demonstrate the performance of the proposed system. The set-up for the experiment in which the task was to pick up objects in a cluttered environment and put them in different target positions for each side is shown in Figure 29: the final success rate is provided in Table 9.

All of the failures were because the objects slipped away to a random direction from original positions right before the robot touched them. The probabilities of failures could be reduced if a bigger container were to be used.

## 6. Conclusions

A completed random bin-picking system for USB packs was proposed and implemented. In particular, this research introduced a robust method that can be used to perform random object picking tasks for planar objects, especially thin objects. The system also serves as a solid basis for random bin-picking tasks with other planar objects in a cluttered environment. The proposed method integrates an instance segmentation-based deep learning approach to classify and locate objects in a scene with a new approach to pick up planar objects by building an appropriate coordination system at the target point of the target object from a single target point in 2D image coordination. Impressive performance was demonstrated using the feature matching technique and the plane segmentation method when handling proper 3D pose estimation. The experimental results showed that the deep learning model could segment each instance in the scene with an average precision of 91.18% while successfully classifying two sides of the object with 100% accuracy. This was a favorable result demonstrating impressive overall accuracy. Furthermore, the proposed appropriate 3D pose estimation achieved accurate results with low average translation and rotation errors. Finally, the pickup success rate exceeded 99%, and the average processing time in each step was less than 0.9 s. Overall, the proposed method provided a stable and reliable solution for managing labor-intensive tasks, otherwise known as random bin-picking tasks, that require repetitiveness and pinpoint accuracy for unstructured and poorly constrained occlusions in heavily cluttered environments.

## Figures and Tables

**Table 1:** Specification for resolution and frame rate of pictures captured by Kinect v1 and Kinect v2.

	Kinect v1		Kinect v2	
	Resolution Pixel × Pixel	Frame Rate (Hz)	Resolution Pixel × Pixel	Frame Rate (Hz)
Color	640 × 480	30	1920 × 1080	30
Depth	320 × 240	30	512 × 424	30
Infrared	320 × 240	30	512 × 424	30

**Table 2:** Number of front and back side instances in the dataset.

	Front	Back	Total
No. of Instances	458	387	845

**Table 3:** Average precision (AP) of the final detection result (%).

Threshold Intersection over Union (IoU)	Average Precision (%)					
	Run	Run	Run	Run	Run	Average
AP@0.5	100	100	100	100	100	100
AP@0.75	100	100	100	100	100	100
AP@0.5:0.95	91.94	90.28	91.21	90.37	92.13	91.18

**Table 4:** Absolute error per pose, with 20 tests per pose for the back side. Results in mm for translation error and in degrees for rotation error.

Pose	Mean Error					
	$\delta T_{x}$	$\delta T_{y}$	$\delta T_{z}$	$\delta R_{x}$	$\delta R_{y}$	$\delta R_{z}$
1	1.208	1.971	2.893	2.277	3.119	1.621
2	0.724	2.319	2.206	2.416	2.870	1.658
3	2.308	2.238	2.094	2.579	2.445	1.114
4	2.852	2.118	2.812	3.164	2.536	1.020
5	0.480	3.506	0.834	2.203	3.744	1.003
6	1.116	4.025	1.431	2.252	2.831	0.689
7	2.583	3.102	4.634	2.187	3.292	0.818
8	0.743	0.813	0.879	1.569	3.548	1.000
9	0.476	1.208	1.490	1.429	4.043	0.744
10	0.791	3.284	2.779	1.557	4.269	0.885
11	3.193	1.106	4.359	1.724	1.178	0.337
12	2.185	2.850	0.888	1.621	1.841	0.759
13	0.734	1.335	1.228	1.641	2.497	1.178
14	2.709	1.025	3.538	1.253	1.437	0.765
15	1.067	0.824	3.534	3.374	1.148	0.707
16	1.981	0.863	3.759	1.276	1.218	0.725
17	0.614	2.337	3.576	1.645	1.571	2.494
18	2.344	1.499	4.968	1.497	0.915	3.522
19	1.318	2.057	2.273	2.219	1.176	4.529
20	1.583	0.701	0.684	2.389	1.221	3.088
21	2.264	1.696	0.615	1.484	1.537	3.904
22	0.909	4.171	0.969	2.551	1.946	4.427
23	3.160	4.061	0.999	2.848	2.023	0.502
24	3.698	3.511	0.881	2.314	2.433	2.101
25	3.941	1.465	1.437	1.733	2.100	2.851
Average	1.799	2.164	2.230	2.048	2.278	1.698

**Table 5:** Absolute error per pose, with 20 tests per pose for the front side. Results in mm for translation error and in degrees for rotation error.

Pose	Mean Error					
	$\delta T_{x}$	$\delta T_{y}$	$\delta T_{z}$	$\delta R_{x}$	$\delta R_{y}$	$\delta R_{z}$
1	0.164	2.150	3.294	2.065	1.223	1.312
2	0.719	1.093	1.451	2.403	1.781	1.059
3	1.878	1.207	1.738	2.335	1.674	1.121
4	1.145	0.405	1.869	3.792	1.314	1.127
5	2.496	1.085	1.140	4.638	2.372	0.691
6	1.050	0.792	1.092	2.000	2.098	0.738
7	0.872	0.126	1.713	3.018	2.883	1.334
8	2.287	1.263	1.211	2.827	2.731	0.878
9	1.101	0.208	1.362	3.006	2.480	0.564
10	0.422	0.829	2.494	2.707	2.992	0.812
11	1.417	0.092	1.873	2.515	3.098	0.387
12	0.465	0.923	2.026	2.095	2.712	1.225
13	0.961	0.346	1.239	2.279	2.866	1.342
14	0.899	0.431	2.266	1.891	1.879	1.867
15	0.224	0.619	1.635	0.703	0.858	0.695
16	0.521	1.185	3.899	1.730	1.342	1.613
17	1.517	0.948	3.520	2.738	1.839	2.549
18	0.430	1.297	3.118	2.602	1.348	3.363
19	1.100	1.257	3.185	2.788	1.660	4.581
20	1.333	0.704	3.707	2.055	1.523	2.772
21	0.972	0.640	2.879	2.150	1.793	3.337
22	1.110	1.654	1.641	2.906	1.457	4.432
23	0.593	0.760	0.900	1.763	1.719	0.527
24	1.509	1.224	1.904	1.869	1.616	2.692
25	2.129	1.158	0.755	2.743	1.650	2.622
Average	1.093	0.896	2.076	2.465	1.956	1.746

**Table 6:** Mean absolute error (MAE) measure for both front and back sides.

MAE Value	Translation Error (mm)			Rotation Error (degrees)			Total Test Number
	$\delta T_{x}$	$\delta T_{y}$	$\delta T_{z}$	$\delta R_{x}$	$\delta R_{y}$	$\delta R_{z}$	*N*
Back	1.799	2.164	2.230	2.048	2.278	1.698	25
Front	1.093	0.896	2.076	2.465	1.956	1.746	25
Average	1.446	1.530	2.153	2.256	2.117	1.722	50

**Table 7:** Real dimensions of the target object.

Object	Length	Width	Depth
USB Flash Drive Pack	101 mm	115 mm	2 mm

**Table 8:** Average processing time in each step of the proposed method.

	Subfunction	Front	Back	Average
Visual Perception	Preprocessing	0.074	0.074	0.074
	Instance Segmentation	0.532	0.532	0.532
Object Pose Estimation	Feature Matching	0.270	0	0.135
	Pose Estimation	0.121	0.121	0.121
Total Processing Time		0.997	0.727	0.862
(In Seconds)				

**Table 9:** Pickup success rate.

Total Trials	Success	Failed	Success Rate
843	840	3	99.64%
